# Assessing Temporal Changes in Microbial Communities in *Hyalomma dromedarii* Collected From Camels in the UAE Using High-Throughput Sequencing

**DOI:** 10.3389/fvets.2022.861233

**Published:** 2022-03-31

**Authors:** Nighat Perveen, Sabir Bin Muzaffar, Ranjit Vijayan, Mohammad Ali Al-Deeb

**Affiliations:** Department of Biology, United Arab Emirates University, Al-Ain, United Arab Emirates

**Keywords:** *Hyalomma dromedarii*, 16S rRNA gene, microbes, temporal pattern, microbe's interaction, high-throughput sequencing, camels, UAE

## Abstract

Ticks (Acari) are ectoparasites of animals that harbor communities of microbes of importance to animal and human health. Microbial communities associated with ticks exhibit temporal patterns of variation in their composition, with different genera dominating at different times of the year. In this study, molecular tools were used to assess the composition of the microbial communities associated with *Hyalomma dromdarii*. Adult ticks were collected every month for 1 year from 25 camels in the UAE. A total of 12 DNA pools were prepared (one pool for each month). We monitored the microbiota of ticks using high-throughput sequencing of the V3–V4 region of the bacterial 16S rRNA gene. A total of 614 operational taxonomic units were produced through *de novo* clustering and belonged to 17 phyla, 30 classes, 46 orders, 118 families, and 222 genera. Fifteen bacterial families were found to be the most abundant. The dominant bacterial communities associated with *H. dromedarii* belonged to the genera *Staphylococcus, Bacillus, Francisella*, and *Corynebacterium*, which were reported with high relative abundance from all months. No significant correlation occurred between the abundance of microbial families or genera in *H. dromedarii* ticks and the ambient temperature. Our findings revealed, for the first time in the UAE, temporal fluctuations of microbial communities in *H. dromedarii* ticks and provided key insights on the interaction between different microbial groups. Moreover, our results contribute to the current understanding of disease development and allow more investigations for potentially pathogenic microbiota.

## Introduction

Ticks transmit pathogens of medical and veterinary importance, which cause serious health issues in humans and considerable economic loss in domestic animals (1). Ticks occupy diverse habitats from tropical areas to the polar region (2). A total of 900 species of ticks have been identified (3). In the last decades, Lyme borreliosis (LB), tick-borne encephalitis (TBE), and other tick-borne diseases (TBDs) have become a growing public health problem across the world (4). In the USA, more than 250,000 human cases of LB have been reported from 2010 to 2019 (https://www.cdc.gov/lyme/stats/tables.html). The incidence of Lyme disease in the USA is expected to increase over 20% in the coming decades due to climate change (5). In Europe, almost all tick bite incidences (90–95%) in humans are caused by *Ixodes* species (4). The camel tick, *Hyalomma dromedarii* (Acari: Ixodidae), is an obligate hematophagous ectoparasite of camels that affects the health of camels (6) and transmits tick-borne diseases to humans. It is the dominant tick species infesting camels in the Middle East and North Africa (MENA) region (7, 8). *Hyalomma dromedarii* has a vital role in the transmission of tick-borne pathogens such as *Theileria, Rickettsia, Francisella*, Crimean-Congo hemorrhagic fever virus (CCHFV), and other viruses (9–15), and poses a serious threat to the camel farming industry. Global climate change, and socio-economic and environmental factors allow ticks and tick-borne pathogens to invade and adapt to new ecological niches (16). The recent reports of the upsurge of tick-borne pathogens/diseases have increased research programs on the tick ecology, vector–host–pathogen interactions, tick genomics, and tick-borne disease epidemiology (17).

High-throughput sequencing technologies have underlined the complexity of the tick microbiota. Tick microbial communities may be composed of multiple pathogens (18) and endosymbionts (19). Microbes that cohabit the midgut of a tick could affect host fitness and its competence (ability to transmit a pathogen) (20). The ecological relationships among these microorganisms may be ranging from beneficial to detrimental (21) resulting in suppression or enhancement of some microbial species (22, 23). Furthermore, these interactions between the microbiota and pathogens are essential to understand because the tick microbiota may sway pathogen colonization and its transmission to the vertebrate host (24). Tick endosymbionts may benefit their hosts by providing nutrients, affecting fitness and reproduction, and immunity (25, 26). In addition, endosymbionts may shape the transmission and infection rate of pathogens. For instance, the occurrence of *Rickettsia bellii* (symbiont) in *Dermacentor andersoni* results in a lower infection rate of *Anaplasma marginale* (pathogen) (27). Moreover, *Rickettsia*-infected *Dermacentor variabilis* revealed greater motility than uninfected ticks, and were significantly faster than *Arsenophonus*-infected ticks, indirectly manipulating disease risk (28). In *Amblyomma maculatum, Candidatus* Midichloria mitochondrii occurrence was recorded in higher concentrations in tissues (midgut, salivary glands, and ovaries) of ticks infected with *Rickettsia parkeri* than in non-infected ticks, which suggested that *Candidatus* Midichloria mitochondrii might support the occurrence of *Rickettsia* sp. in *A. maculatum* (29). Therefore, such inter-microbial species interactions suggest that endosymbionts may affect microbial community structure and disease transmission (25, 30).

Tick microbiota may be influenced by temporal or spatial scales, and it is important to consider temporal patterns in studies on tick microbial communities to understand tick-borne microbe ecology and microbiota as well as pathogen interactions (31). Microbial infections are pervasive in animal and human populations in healthy ecosystems (32). A limited number of studies on the microbial community structure of *H. dromedarii* (22, 33–35) pointed to complex microbial assemblages in dwelling ticks comprising endosymbionts, commensals, and pathogens. Because in the UAE *H. dromedarii* ticks exist in the desert ecosystem under harsh environmental conditions, especially the extreme summer heat and drought, we hypothesize that their microbial species composition has gone way off in terms of the types of typical taxa found in other tick species or *H. dromedarii* in temperate environments. In addition, it is known that the geographical location and the environment tend to dictate the type of microbiota in an organism, and hence we expect a level of microbes' adaptation to the desert environment, which may lead to different microbiota compositions. Thus, these ticks in the desert ecosystem may not cluster, in terms of the microbes, where most of the ticks of the same species cluster in other parts of the world. Tick microbes could play significant roles in the maintenance of tick populations on camels under harsh environmental conditions. Therefore, the present study aims to characterize the fluctuation of microbial diversity in *H. dromedarii* over a year using high-throughput sequencing. Furthermore, it aims to assess microbes' response to season/temperature.

## Materials and Methods

### Sample Collection and Tick Identification

The study was conducted on one camel farm over a year (March 2019–February 2020) in Al Ain, in the emirate of Abu Dhabi, UAE. Ticks were collected from 25 camels each month in sterile plastic tubes (50 ml), kept in an icebox, and shifted to the animal ecology and entomology laboratory, UAE University, where ticks were stored at −80°C until DNA extraction. Ticks were collected according to the protocol approved by the Animal Research Ethics Committee (A-REC) of the UAE University (ethical approval no. ERA_2019_5953). The identification of *H. dromedarii* ticks was confirmed morphologically using taxonomic keys (6). In addition, ticks were identified at the molecular level using 16S rRNA gene with the primers 16S+1 and 16S−1 (5′-CTGCTCAATGATTTTTTAAATTGCTGTGG-3′ and 5′-CCGGTCTGAACTCAGATCAAGT-3′, respectively). The details of sample processing, DNA extraction, and PCR conditions had been mentioned elsewhere (36). The mean monthly temperature in degrees Celsius (°C) was obtained from the nearest meteorological station.

### Extraction of Genomic DNA and Pooling

Only partially engorged females of *H. dromedarii* were used for DNA extraction where five ticks from each month were randomly selected. Before DNA extraction, ticks were washed by using ethanol and distilled water following a protocol (37). Each selected individual tick was homogenized using a sterile Kimble Kontes pellet pestle (Thermo Fisher, Waltham, MA) in a sterile 1.5-ml microcentrifuge tube. Genomic DNA was extracted from individual ticks using QIAamp Tissue Kit (Qiagen, Hilden, Germany) following the manufacturer's protocol. A spectrophotometer (Nano Drop ND-1000, Erlangen, Germany) was used to measure the concentration and quality of DNA. In addition, the quality of DNA was checked on 1.5% agarose gel. Genomic DNAs from five ticks were pooled and a total of 12 pools were prepared (one pool for each month) and then the DNA was stored at −80°C in the freezer until further use.

### High-Throughput Sequencing and Bioinformatics

To determine the temporal pattern of the microbes in *H. dromedarii*, 16S rRNA gene-based analysis was performed. For high-throughput sequencing, a total of 12 DNA pools from 12 months were sent for sequencing to Macrogen Inc. (Seoul, South Korea). DNA pools for ten samples (representing 10 months) passed the quality check for NGS, while the pools from August 2019 and February 2020 did not pass. A pair of primers (forward primer: 5′-TCGTCGGCAGCGTCAGATGTGTATAAGAGACAGCCTACGGGNGGCWGCAG-3′, reverse primer: 5′-GTCTCGTGGGCTCGGAGATGTGTATAAGAGACAGGACTACHVGGGTATCTAATCC-3′) (38), was used to amplify the hypervariable V3 V4 region, and PCR was performed by using the Herculase II Fusion DNA polymerase Nextera XT Index Kit V2. Furthermore, Illumina's MiSeq platform was used to perform the sequencing with a read length of 301 bp. Reads containing more than 30 bases with a PHRED score of <10 were filtered out. Paired-end sequences in FASTQ format were merged using fast length adjustment of short reads (FLASH) version 1.2.11 (39). The merged reads were clustered into operational taxonomic unites (OTUs) with CD-HIT-OTU (40) using the default options. The preprocessing stage of CD-HIT-OTU workflow filtered low-quality reads, trimmed long tails, identified chimeric reads, and subsequently clustered the reads into OTUs with a 97% identity cutoff. Finally, taxonomic assignment of OTUs was performed using the assign_taxonomy.py script of QIIME1.9.1 (41). The assignment was based on Basic Local Alignment Search Tool (BLAST) (42) searches in the Ribosomal Database Project (RDP; http://rdp.cme.msu.edu/) and National Center for Biotechnology Information (NCBI) 16S rRNA database. Taxonomic abundance count was analyzed to calculate abundance ratios at the phylum, class, family, and genus levels in Microsoft Excel. Illustrations were produced using GraphPad Prism GraphPad Prism 8.3.1 for Windows (San Diego, CA, USA, www.graphpad.com) and PAST 5.27 Paleontological statistics software package (43).

### Data Accessibility Statement

The high-throughput data of 16S rRNA gene-based analysis of the present study were deposited in the NCBI Sequence Read Archive under the BioProject ID: PRJNA763903.

### Statistical Analyses

We calculated the richness, Shannon–Wiener Index, and the Index of Dominance to characterize patterns of diversity of bacterial communities in *H. dromedarii* over a year using the PAST 5.27 Paleontological statistics software package (43). We then performed Principal Coordinates Analysis (PCoA) to better evaluate these diversity patterns. The OTUs of each genus were entered and the samples were classified by months. Eigenvalues were examined and the magnitude of variation explained by individual principle coordinates was determined (44). Associations between different genera were estimated through Pearson's correlation coefficient (*r*) (45). Furthermore, we performed stepwise regression analysis with backward selection with the genera, which only showed significant correlations (45). For all tests, the value of significance level (α) was set at 0.05. In addition, associations between the abundance (%) of microbial families or genera in *H. dromedarii* ticks and the ambient temperature were estimated through Pearson's correlation coefficient (*r*).

## Results

### Microbiota Diversity and Composition

From the current dataset, we attained 563,688 sequence reads ranging from 44,327 to 73,697 sequence reads, with an average of 56,368.8 reads. Taxonomic classification was accomplished by using raw reads after quality filtering of sequences. A total of 614 OTUs were produced through *de novo* clustering (OTUs, clustered at 97% similarity) that belonged to 17 phyla, 30 classes, 46 orders, 118 families, and 222 genera. After taxonomic profiling, the presence of nine abundant phyla was confirmed. *Firmicutes* and *Actinobacteria* were found the most abundant phyla whereas *Acidobacteria* and *Spirochaetes* had the least abundance (Supplementary Table 1).

Eleven bacterial classes were abundant out of 30 total classes. *Gammaproteobacteria, Bacilli*, and *Actinobacteria* were the most abundant in all months; however, *Gammaproteobacteria* was found the most dominant (80.7%) in September 2019. The *Alphaproteobacteria* was found as the dominant class (79%) in November 2019, and *Bacilli* was abundant (60.5%) in December 2019, while *Actinobacteria* was abundant (55%) in January 2020 (Supplementary Table 2). Out of 46 orders, 14 orders were abundant in ticks collected over a year (Supplementary Table 3). The class *Bacillales* was found to be the most dominant (62%) in July 2019, while *Actinomycetales* was dominant (54.7%) in January 2020 and *Enterobacteriales* (33.9%) in April 2019.

**Figure 1:**
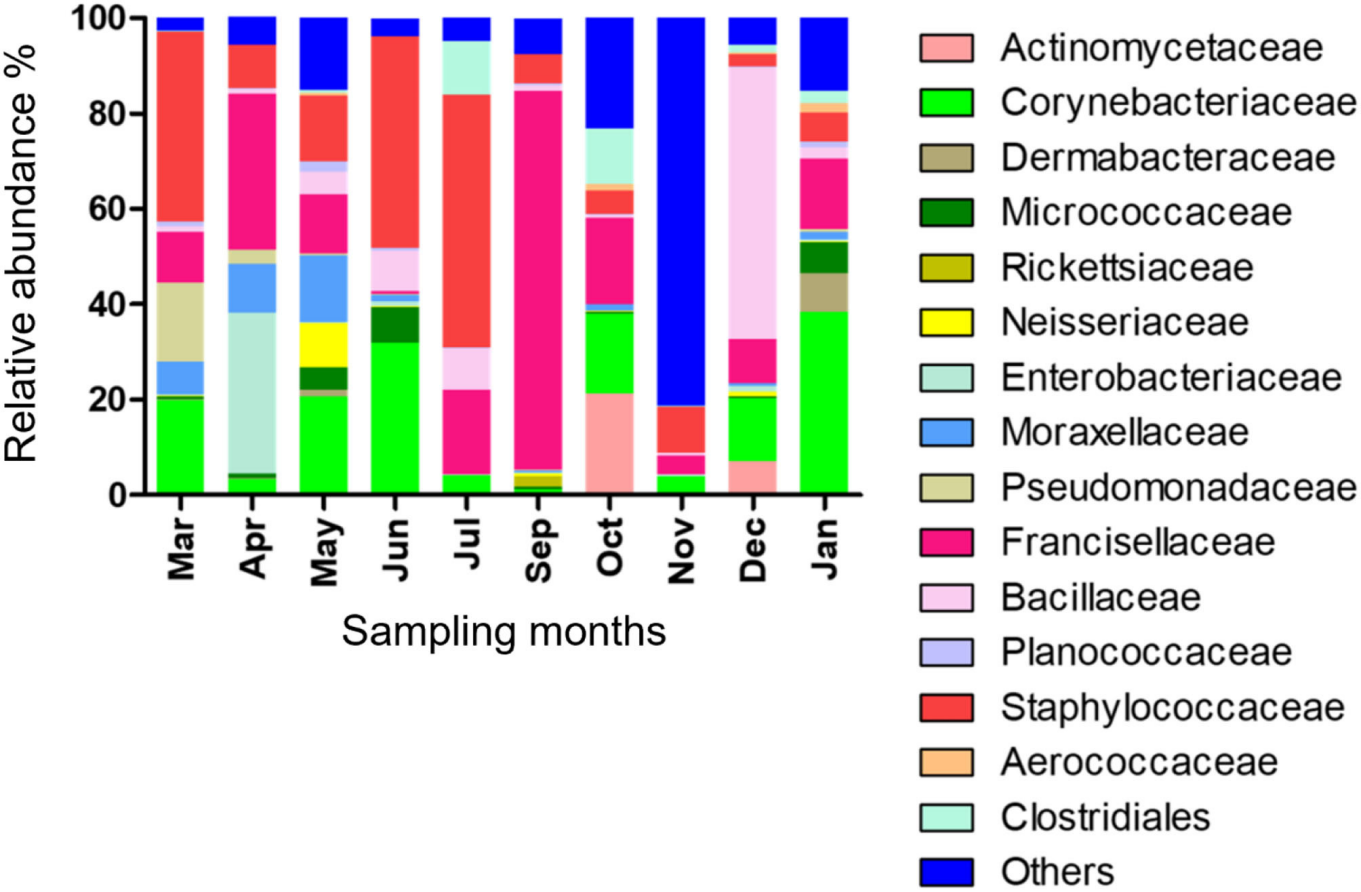
Microbial families detected in *H. dromedarii* partially engorged female ticks from 10 months.

Fifteen bacterial families were found abundant (Supplementary Table 4). *Staphylococcaceae* was reported with the highest relative abundance (53.1, 44.2, and 39.9%) in July, June, and March 2019, respectively, and *Bacillaceae* was reported with high relative abundance (57%) in December 2019. *Francisellaceae* and *Corynebacteriaceae* were found with high relative abundance in all months; however, *Francisellaceae* showed the highest relative abundance in September 2019 and *Corynebacteriaceae* showed the highest relative abundance in January 2020 (Figure 1; Supplementary Table 4).

**Figure 2:**
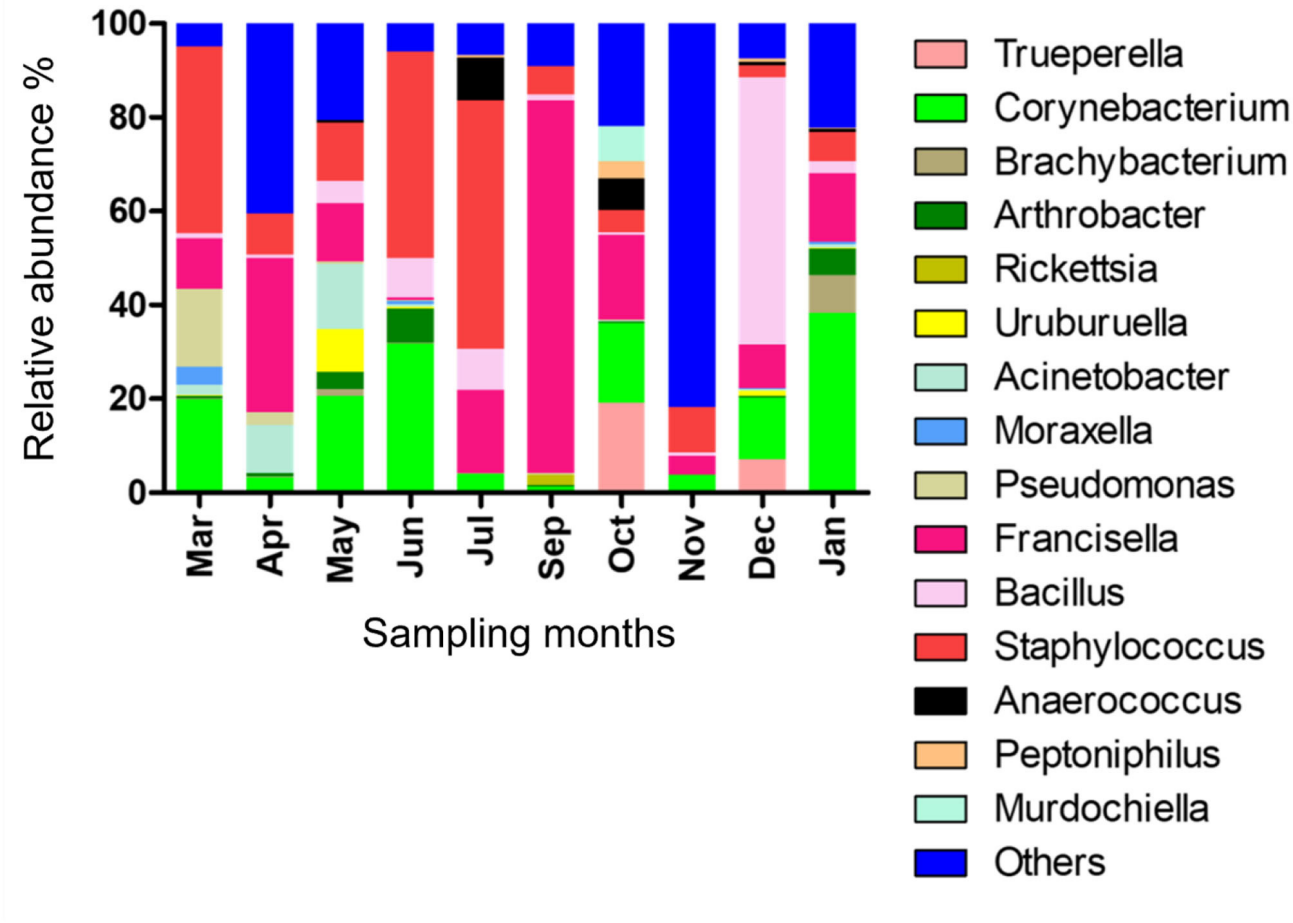
Microbial genera detected in *H. dromedarii* partially engorged female ticks from 10 months.

The relative abundance of bacterial genera in the microbiota of *H. dromedarii* over a year was highly variable among months. *Staphylococcus, Bacillus, Francisella*, and *Corynebacterium* were the most common genera reported with high relative abundance from all months; however, *Francisella* was found the most abundant 79.4% in September 2019, *Bacillus* (57%) in December 2019, *Staphylococcus* (53.1%) in July 2019, and *Corynebacterium* (38.2%) in January 2020. *Trueperella* was reported only in 3 months, June, October, and December 2019, and *Murdochiella* was reported only in 2 months, October 2019 and January 2020, however, both reported with high relative abundance in October (19.2%) and (7.5%), respectively. *Rickettsia* (2.1%) was reported only in September 2019 (Figure 2; Supplementary Table 5).

### Temporal Dynamics of the Microbiota

**Figure 3:**
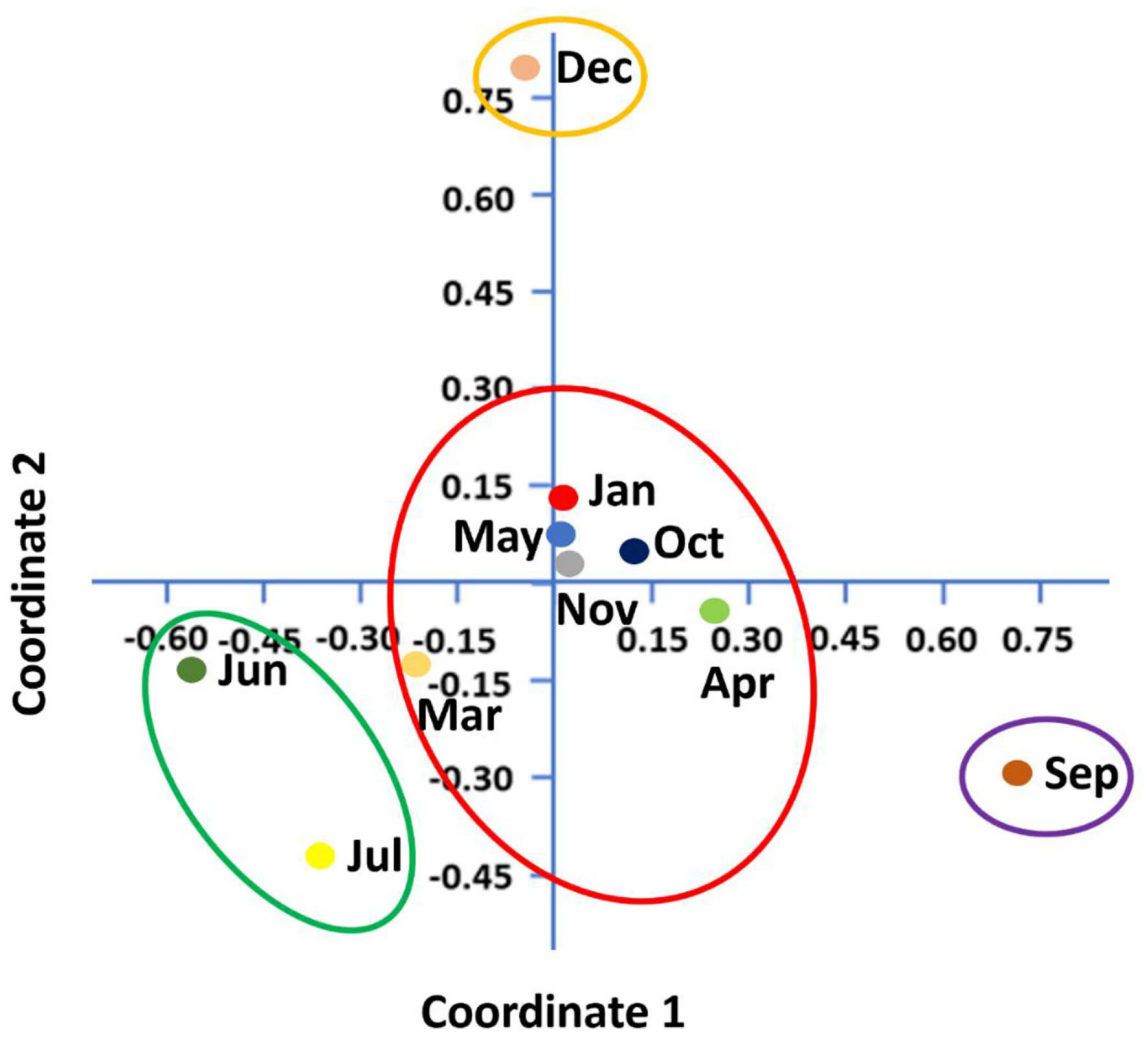
Principal coordinates analysis (PCoA) shows microbial diversity between months in *H. dromedarii* partially engorged female ticks.

**Figure 4:**
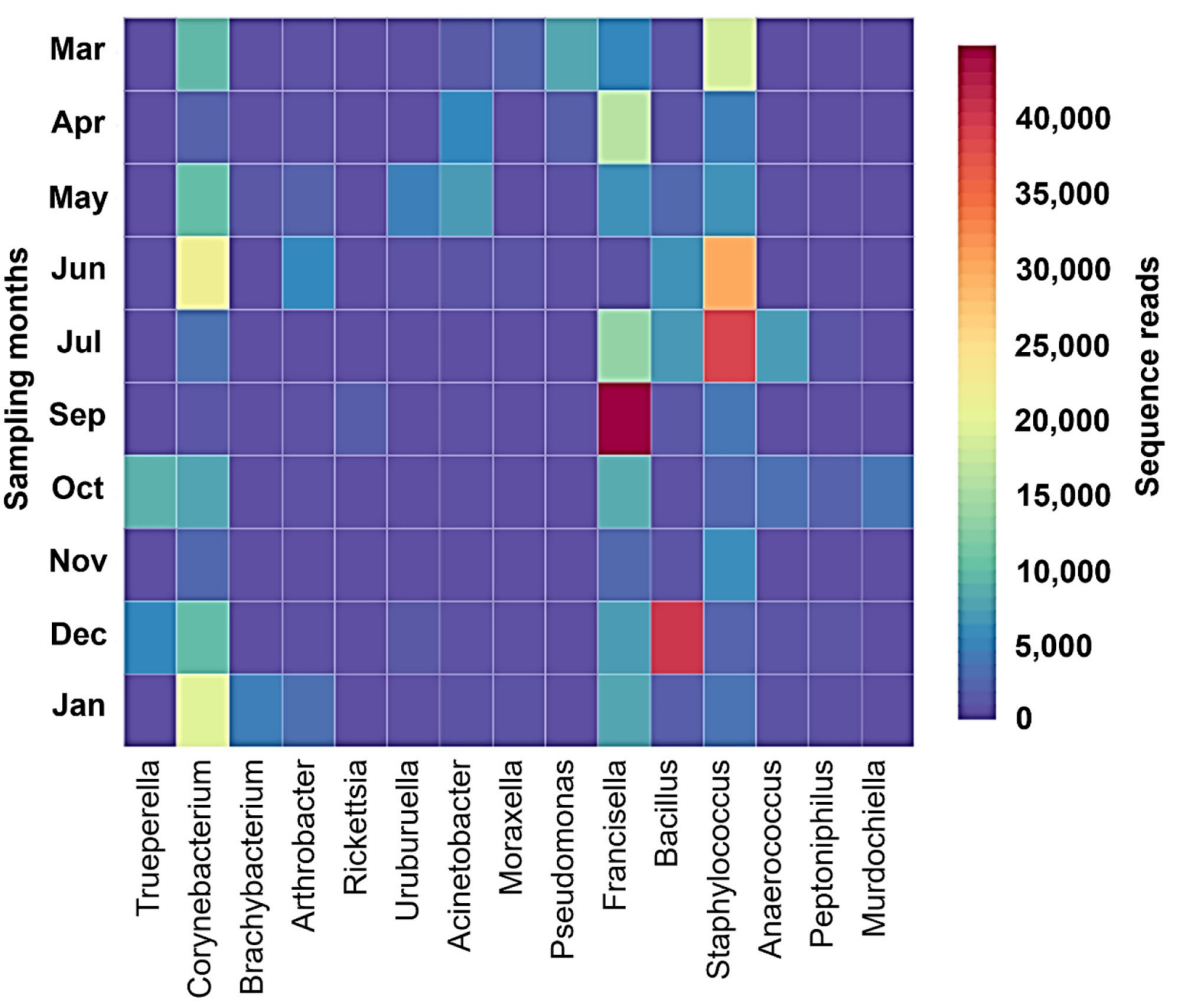
Matrix plot shows dominant genera in different months in *H. dromedarii* partially engorged female ticks.

Shannon–Wiener index values differed significantly between different months indicating that microbial communities changed significantly during the study period. Diversity indices and diversity *t*-test showed that richness and evenness of genera differed significantly between tick samples collected in different months. For example, the richness of genera differed significantly between September and December (6.46 in September vs. 3.72 in December; two-sample paired *t*-test, *p* < 0.01). The Shannon–Wiener index differed significantly between September and December (0.73 [95% confidence interval: 0.72–0.74] vs. 1.44 [95% confidence interval: 1.43–1.45], respectively; two-sample paired *t*-test, *p* < 0.05). The Index of Evenness was significantly lower in September than in December (0.10 vs. 0.18, respectively; two-sample paired *t*-test, *t* = −88.54, *p* < 0.01). In addition, the Index of Dominance was significantly different between September and December (0.72 vs. 0.39, respectively; two-sample paired *t*-test, *p* < 0.01). Furthermore, the Shannon–Wiener index did not differ significantly between June and July (1.44 [95% confidence interval: 1.43–1.45] vs. 1.35 [95% confidence interval: 1.34–1.36], respectively; two-sample paired *t*-test, *p* > 0.05). The Index of Dominance did not differ significantly between June and July (0.33 vs. 0.36, respectively; two-sample paired *t*-test, *p* > 0.01). Principal Coordinates Analysis showed that coordinates 1, 2, and 3 accounted for over 88% of the variation based on cumulative Eigenvalues and the first two coordinates accounted for over 67% of the variation. There was a separation between the microbial communities in June and July, September, December, and other months (March, April, May, October, November, and January) (Figure 3). Furthermore, the Matrix plot confirmed the heavy load of *Bacillus* in December and *Francisella* in September (Figure 4). These 2 months are distant from others in PCoA likely because of the dominance of these different genera. The closer clustering of June and July in the PCoA (Figure 3) was possibly due to the dominant genus *Staphylococcus* (Figure 4).

### Patterns of Microbial Association Within *H. dromedarii*

**Figure 5:**
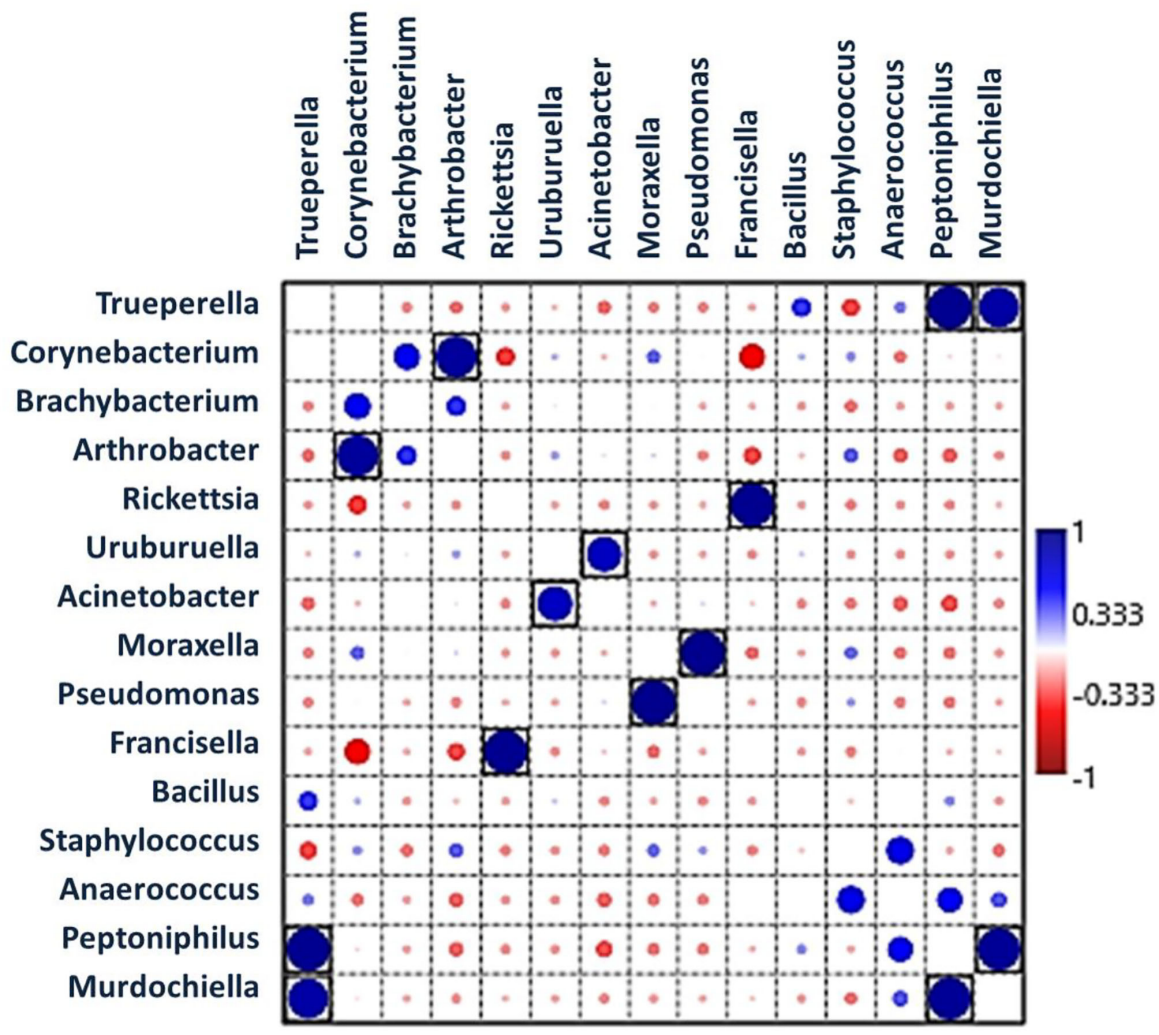
Pearson's correlation coefficients indicate associations between bacterial genera showing significantly positive interactions (large dark blue circles) and negative interactions (red circles). Black boxes denote *p* < 0.05.

Pearson's correlation coefficients (*r*) pointed out that several microbial genera were significantly correlated with each other (*p* < 0.05, boxed circles) (Figure 5; Supplementary Table 6). *Francisella* was significantly positively correlated with *Rickettsia*, whereas *Corynebacterium* was significantly positively correlated with *Arthrobacter*. *Pseudomonas* was significantly positively correlated with *Moraxella*, and *Peptoniphilus* was correlated with *Trueperella* and *Murdochiella*. In addition, *Acinetobacter* had a positive correlation with *Uruburuella*. However, *Francisella* showed a negative correlation with *Corynebacterium*.

### Effect of Ambient Temperature on Microbial Communities

**Figure 6:**
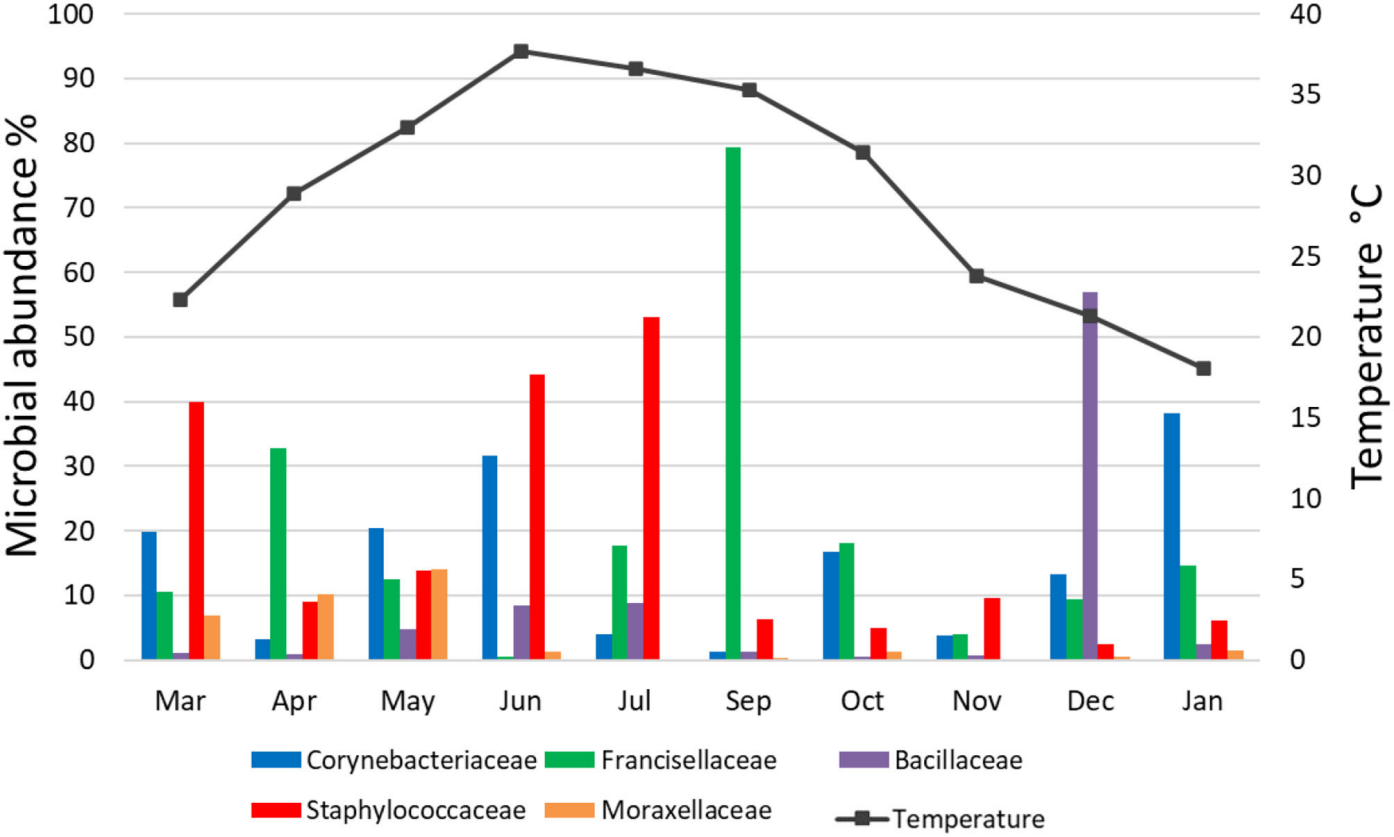
Variation in the relative abundance of bacterial families in relation to temperature in *H. dromedarii* partially engorged female ticks throughout the study.

**Figure 7:**
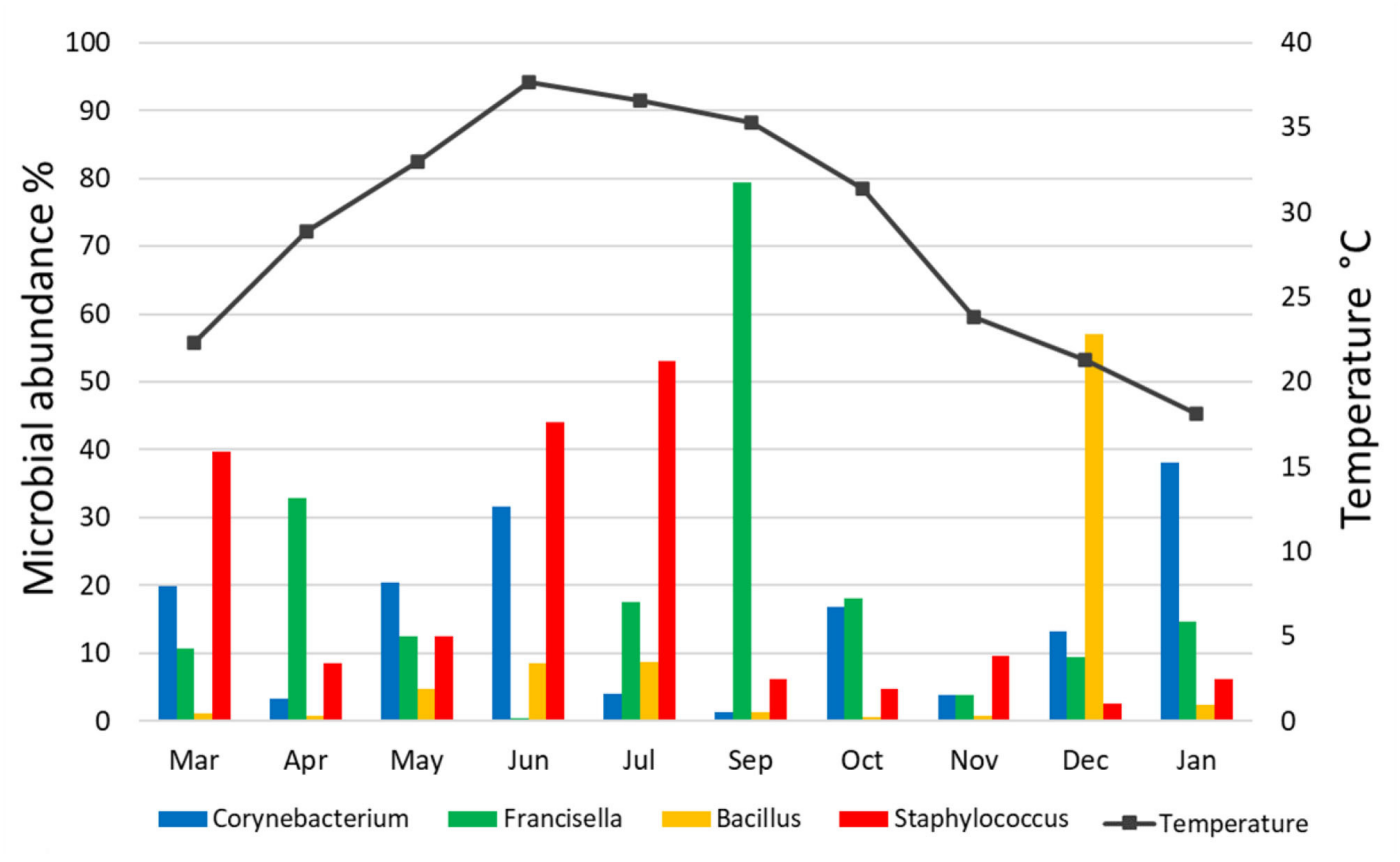
Variation in the relative abundance of bacterial genera in relation to temperature in *H. dromedarii* partially engorged female ticks throughout the study.

There was no significant correlation, measured by Pearson's correlation coefficient (*r*), between the abundance (%) of microbial families (Figure 6) or genera (Figure 7) in *H. dromedarii* ticks and the ambient temperature (*p* < 0.05). Among the hot months, May had the highest genus richness (12 genera) and six genera (*Francisella, Brachybacterium, Bacillus, Arthrobacter, Corynebacterium*, and *Staphylococcus*) were the most dominant among these months. However, in the cold months, January had the highest genus richness (12 genera) and also six genera (*Francisella, Bacillus, Arthrobacter, Corynebacterium, Acinetobacter*, and *Staphylococcus*) were the most dominant during this time of the year. However, the genus *Brachybacterium* in the hot months was replaced by *Acinetobacter* in the cold months.

## Discussion

We investigated the microbiota of *H. dromedarii* over a year and detected 614 OTUs (belonged to 222 genera), which were more than previous studies in which 371 OTUs belonged to 202 genera (22) and 546 OTUs belonged to 114 genera (34). However, it should be noted that these studies were not focused to determine temporal patterns of microbial communities in *H. dromedarii*. *Firmicutes, Proteobacteria*, and *Actinobacteria* were the abundant phyla found in the present dataset and notably, *Firmicutes* was the most abundant one in all months. These results were slightly different from previous studies that reported all three phyla; however, they found that *Proteobacteria* was the most abundant phylum (22, 33, 34).

*Gammaproteobacteria, Alphaproteobacteria*, and *Bacilli* were the most abundant classes in the present study and our results were almost similar to the findings of Khoo et al. (46), who reported *Gammaproteobacteria, Alphaproteobacteria, Actinobacteria, Bacilli*, and *Deltaproteobacteria* from *Haemaphysalis* ticks in Malaysia. In addition, our findings were in agreement with Karim et al. (47), who identified six dominant bacterial classes such as *Bacilli, Gammaproteobacteria, Betaproteobacteria, Clostridia, Alphaproteobacteria*, and *Actinobacteria* from ticks collected from livestock in Pakistan.

We identified *Staphylococcaceae, Bacillaceae, Francisellaceae*, and *Corynebacteriaceae* with high relative abundance in ticks and the current pattern of bacterial families was different from the previous studies (22, 33). In similar studies on microbes of the *Amblyomma maculatum*, Budachetri et al. (48) mentioned *Francisellaceae, Enterobacteriaceae*, and *Rickettsiaceae* as abundant bacterial families detected in field-collected ticks. These variations in bacterial families' patterns may be due to different biotic and abiotic factors, for instance, sampling locations and season of sampling, environmental factors and microclimatic conditions, farming practices, and hosts (breeds, age, and sex).

*Staphylococcus, Bacillus, Francisella*, and *Corynebacterium* were the most common genera in all months, and the results were a little different from the results of our previous finding where *Acinetobacter* and *Corynebacterium* were the most common genera in 2010 and *Francisella* in 2019 (22). Focusing on *Francisella*, the sequences have already been detected in all studies on *H. dromedarii* and a study on *Hyalomma anatolicum* characterizing microbial composition in the MENA region (22, 33–35, 49). In the present study, *Francisella* was detected with high relative abundance in September followed by April and October. Moreover, it was found with the lowest relative abundance in November. However, no significant correlation was determined between its abundance in different months and temperature. *Francisella* in the MENA region had been characterized as *Francisella*-Like Endosymbiont (FLE) (13, 35, 50) that may be the source of several vitamins and cofactors for ticks lacking in hosts' blood meals, and improve tick fitness (51). This bacterium can be transmitted in tick generations via vertical transmission (51). *Francisella* had been detected in several tick species in mutualistic forms (30). However, similarities between pathogenic and mutualistic *Francisella* in phylogenetics suggest frequent shifts from non-pathogenic forms to pathogenic (30, 52). Tick symbionts also affect pathogen colonization and transmission to the vertebrate host, for instance, FLEs have favored the establishment of *Francisella novicida* in *Dermacentor andersoni* (27). On the other hand, *Rickettsia* is a maternally inherited bacterium in arthropods and was found previously in a study on temporal patterns of microbial communities in *Ixodes ricinus* (23). *Rickettsia* occurs in nature as endosymbionts and pathogens. Sometimes pathogenic microbial species circulate in tick hosts in a benign form. While determining the pathogenicity of *Rickettsia* species (30), it was previously revealed that species presumed to be symbionts, for example, *Rickettsia helvetica* and *Rickettsia slovaca* (53), were actually the pathogenic ones (54). Furthermore, in the present study, it was detected (2.13%) only in September, where we noticed the highest relative abundance of *Francisella* (79.42%) in the samples. In addition, a positive association of *Francisella* with *Rickettsia* in our dataset (Figure 4) may suggest a synergistic interaction between them (55). Moreover, the co-existence of tick endosymbionts and multiple pathogens in the gut of the tick could influence the tick vectorial capacity by affecting tick-borne pathogens' establishment and transmission to humans and animals and change the tick-borne disease's dynamics (24, 56). In the UAE, *H. dromedarii* was found as the most prevalent tick throughout the year on camels (57). The high abundance of endosymbionts such as *Francisella* and *Rickettsia* (maternally inherited bacteria), microclimatic conditions, and high abundance of vertebrate hosts due to widespread camel farming might be supporting the camel tick population even under harsh climatic conditions.

In the present study, *Pseudomonas, Bacillus*, and *Acinetobacter* that were identified in camel ticks had been reported previously in tick species (37, 55, 58). Ticks could acquire these bacteria through openings, for example, mouth, spiracles, or anal pore (52). Our dataset was subjected to contaminant filtering and quality control to reduce contamination in OTUs. We performed the surface sterilization of the ticks using ethanol, which is a good method of surface sterilization. However, according to some studies, it is not a hundred percent efficient at removing bacterial DNA from the tick cuticle as bleach (59). Nonetheless, in studies assessing the microbial communities, environmental bacteria were found even after using bleach (60). Therefore, some environmental bacteria might be encountered on ticks after using any surface sterilization method, but at very low abundance. Interestingly, we detected a significant positive association of *Pseudomonas* with *Moraxella*, which could suggest that one bacterium may influence the other. Such associations likely indicate that co-infected ticks with multiple pathogens might intensify the clinical complexity of diseases, which may result in serious threats to human and animal health (61). In addition, *Staphylococcus* was found with high relative abundance in June and July as compared with other months. Furthermore, *Corynebacterium* and *Bacillus* were also detected in these two months. Microbial interactions in ticks may influence pathogen characteristics and its transmission (62), for example, non-pathogenic rickettsiae in *Dermacentor andersoni* serves as a limiting factor for the distribution of *Rickettsia rickettsia* and rickettsial diseases (63). Coinfections in ticks affect pathogen acquisition, transmission, and host infection risk (64). Therefore, understanding and knowledge of co-occurring pathogens are important for controlling tick-borne zoonotic diseases.

To illustrate the temporal patterns/dynamics in the dataset, principal component analysis was performed. It provided evidence that the *H. dromedarii* microbial communities were grouped into two clusters. Bacterial communities in the summer months, June and July, grouped into one cluster and the rest of the months grouped into the second cluster; however, bacterial communities in September and December were at distance in the second cluster. This temporal pattern may suggest that during the summer, temperature, humidity, and host factors (health, behavior, sex, age) might impact the dynamics of microbial communities' structure in ticks. So far, the effect of environmental conditions on the microbes' diversity in ticks is an understudied area. One study has shown that bacterial community composition changes significantly over time; however, the main factor was the host identity and not the environmental conditions (65). Similarly, the results of the current study are in agreement with this finding and no significant correlation was found between the abundance (%) of microbial families or genera in *H. dromedarii* ticks and the ambient temperature. Overall, there was no clear pattern in the temporal changes of the microbial communities in the current study. Interestingly, the four most abundant genera had peaks at two different temperatures, where *Francisella* and *Staphylococcus* peaked during hot months (September and July, respectively), whereas *Bacillus* and *Corynebacterium* peaked in colder months (December and January, respectively). Furthermore, we noticed that during the hot months, May had the highest richness in which six dominant genera were the most dominant taxa, while in the cold months, January had the highest richness with six dominant genera. Although the number of the dominant genera did not change irrespective of the ambient temperature, the genus *Brachybacterium* in the hot months was replaced by *Acinetobacter* in the cold months. It appears that tick microbes and the association among them shape the structure of microbiota of different tick species and ultimately affect the tick-borne diseases ecology and epidemiology. In addition, knowledge and understanding of tick-borne microbe interactions inside the tick and the effect of abiotic factors are crucial for planning and developing new strategies to control ticks. It is worth noting that future studies should consider assessing beta diversity among tick microbial communities by including more than one sampling location. Accordingly, valuable information is likely to come from this type of study.

## Conclusion

Our study is the first to provide a record of the temporal microbial communities associated with *H. dromedarii* ticks in the UAE and MENA region. Our data demonstrated that changes occurred in the abundance of bacterial groups over time and that some genera maintained high relative abundance all the time while conversely certain genera reached zero or low levels in some months. The findings of this study could help to improve our current knowledge of the changes in *H. dromedarii* microbiota. Moreover, there is a need to conduct more studies in the future to fully characterize the temporal changes of the microbiota in this important tick species.

## Data Availability Statement

The datasets presented in this study can be found in online repositories. The names of the repository/repositories and accession number(s) can be found at: https://www.ncbi.nlm.nih.gov/genbank/, PRJNA763903.

## Ethics Statement

The animal study was reviewed and approved by Animal Research Ethics Committee (A-REC) of UAE University (ethical approval no.: ERA_2019_5953).

## Author Contributions

MA-D, SM, and NP conceived and designed the study and performed the statistical analyses. NP collected and sorted the ticks. NP and MA-D identified the ticks, did the molecular laboratory work, and prepared the graphs. NP, RV, SM, and MA-D analyzed the data and wrote the article. MA-D and SM acquired funding. MA-D supervised and managed the project. All authors have read and approved the final article.

## Funding

The funding of this study was provided by the UAE University through UPAR Grant # G00002604.

## Conflict of Interest

The authors declare that the research was conducted in the absence of any commercial or financial relationships that could be construed as a potential conflict of interest.

## Publisher's Note

All claims expressed in this article are solely those of the authors and do not necessarily represent those of their affiliated organizations, or those of the publisher, the editors and the reviewers. Any product that may be evaluated in this article, or claim that may be made by its manufacturer, is not guaranteed or endorsed by the publisher.
