# Supplementary material for: Assessing Temporal Changes in Microbial Communities in Hyalomma dromedarii Collected From Camels in the UAE Using High-Throughput Sequencing
Source: Front Vet Sci. 2022 Mar 31;9:861233. doi: 10.3389/fvets.2022.861233 (PMC9008585; doi:10.3389/fvets.2022.861233)

Supplementary Material

**Table S1.** Microbial phyla (presence in %) detected in *H. dromedarii* over a year.

| **Phylum** | **19.Mar** | **19.Apr** | **19.May** | **19.Jun** | **19.Jul** | **19.Sep** | **19.Oct** | **19.Nov** | **19.Dec** | **20.Jan** |
| --- | --- | --- | --- | --- | --- | --- | --- | --- | --- | --- |
| Acidobacteria | 0.16% | 0.00% | 0.00% | 0.00% | 0.00% | 0.00% | 0.27% | 0.00% | 0.00% | 0.10% |
| Actinobacteria | 20.84% | 4.83% | 32.28% | 40.42% | 4.60% | 2.31% | 40.50% | 4.06% | 22.77% | 55.04% |
| Bacteroidetes | 0.00% | 0.88% | 0.29% | 0.57% | 0.20% | 0.87% | 2.01% | 0.58% | 0.99% | 1.80% |
| Chloroflexi | 0.02% | 0.00% | 0.05% | 0.01% | 0.14% | 0.00% | 2.27% | 0.00% | 0.01% | 0.00% |
| Planctomycetes | 0.00% | 0.00% | 0.01% | 0.00% | 0.00% | 0.00% | 0.07% | 0.07% | 0.00% | 0.03% |
| Proteobacteria | 34.82% | 80.60% | 38.47% | 4.46% | 20.75% | 84.86% | 21.68% | 83.73% | 13.58% | 21.03% |
| Spirochaetes | 0.00% | 0.29% | 0.01% | 0.00% | 0.11% | 0.00% | 0.00% | 0.00% | 0.00% | 0.00% |
| Cyanobacteria/Chloroplast | 0.00% | 0.05% | 0.06% | 0.07% | 0.15% | 0.16% | 0.12% | 0.13% | 0.00% | 0.01% |
| Firmicutes | 44.10% | 12.86% | 28.69% | 54.47% | 73.90% | 11.30% | 32.87% | 11.28% | 62.50% | 21.71% |

**Table S2.** Microbial classes (presence in %) detected in *H. dromedarii* over a year.

| **Class** | **19.Mar** | **19.Apr** | **19.May** | **19.Jun** | **19.Jul** | **19.Sep** | **19.Oct** | **19.Nov** | **19.Dec** | **20.Jan** |
| --- | --- | --- | --- | --- | --- | --- | --- | --- | --- | --- |
| Actinobacteria | 20.84% | 4.83% | 32.28% | 40.42% | 4.60% | 2.31% | 40.50% | 4.06% | 22.77% | 55.04% |
| Bacteroidia | 0.00% | 0.84% | 0.06% | 0.01% | 0.18% | 0.17% | 0.20% | 0.52% | 0.99% | 0.76% |
| Sphingobacteriia | 0.00% | 0.01% | 0.05% | 0.02% | 0.02% | 0.23% | 1.57% | 0.01% | 0.00% | 0.54% |
| Thermomicrobia | 0.02% | 0.00% | 0.04% | 0.01% | 0.00% | 0.00% | 1.10% | 0.00% | 0.00% | 0.00% |
| Alphaproteobacteria | 0.12% | 0.30% | 0.90% | 0.24% | 0.27% | 2.96% | 1.48% | 79.14% | 0.86% | 1.46% |
| Betaproteobacteria | 0.20% | 0.20% | 9.66% | 0.39% | 0.00% | 1.01% | 0.18% | 0.15% | 1.50% | 1.31% |
| Gammaproteobacteria | 34.50% | 80.00% | 27.86% | 3.83% | 20.48% | 80.65% | 20.02% | 4.44% | 11.04% | 18.27% |
| Bacilli | 43.60% | 11.46% | 24.89% | 54.31% | 62.60% | 9.29% | 7.70% | 10.87% | 60.47% | 13.22% |
| Clostridia | 0.50% | 1.23% | 3.47% | 0.15% | 11.30% | 1.77% | 23.42% | 0.36% | 1.94% | 6.55% |
| Erysipelotrichia | 0.00% | 0.13% | 0.32% | 0.01% | 0.00% | 0.25% | 0.08% | 0.04% | 0.04% | 1.56% |
| Negativicutes | 0.00% | 0.00% | 0.01% | 0.00% | 0.00% | 0.00% | 1.67% | 0.00% | 0.00% | 0.37% |

**Table S3.** Microbial orders (presence in %) detected in *H. dromedarii* over a year.

| **Order** | **19.Mar** | **19.Apr** | **19.May** | **19.Jun** | **19.Jul** | **19.Sep** | **19.Oct** | **19.Nov** | **19.Dec** | **20.Jan** |
| --- | --- | --- | --- | --- | --- | --- | --- | --- | --- | --- |
| Actinomycetales | 20.84% | 4.83% | 32.28% | 40.42% | 4.31% | 2.26% | 39.54% | 3.96% | 22.77% | 54.73% |
| Bacteroidales | 0.00% | 0.84% | 0.06% | 0.01% | 0.18% | 0.17% | 0.20% | 0.52% | 0.99% | 0.76% |
| Sphingobacteriales | 0.00% | 0.01% | 0.05% | 0.02% | 0.02% | 0.23% | 1.57% | 0.01% | 0.00% | 0.54% |
| Sphaerobacterales | 0.02% | 0.00% | 0.04% | 0.01% | 0.00% | 0.00% | 1.10% | 0.00% | 0.00% | 0.00% |
| Rickettsiales | 0.00% | 0.00% | 0.00% | 0.00% | 0.00% | 2.13% | 0.00% | 0.00% | 0.00% | 0.00% |
| Neisseriales | 0.12% | 0.02% | 9.14% | 0.34% | 0.00% | 0.80% | 0.02% | 0.00% | 1.13% | 0.38% |
| Enterobacteriales | 0.19% | 33.85% | 0.14% | 1.04% | 0.05% | 0.16% | 0.14% | 0.34% | 1.20% | 0.33% |
| Chromatiales | 0.00% | 0.00% | 0.01% | 0.01% | 2.52% | 0.00% | 0.00% | 0.00% | 0.00% | 0.62% |
| Pseudomonadales | 23.48% | 13.09% | 14.55% | 1.46% | 0.13% | 0.45% | 1.36% | 0.02% | 0.46% | 2.25% |
| Thiotrichales | 10.67% | 32.86% | 12.43% | 0.50% | 17.63% | 79.42% | 18.12% | 3.94% | 9.37% | 14.66% |
| Bacillales | 42.01% | 10.28% | 23.04% | 53.42% | 62.08% | 8.17% | 5.69% | 10.38% | 59.62% | 10.16% |
| Lactobacillales | 1.59% | 1.17% | 1.85% | 0.88% | 0.51% | 1.12% | 2.01% | 0.49% | 0.85% | 3.06% |
| Clostridiales | 0.50% | 1.23% | 3.46% | 0.15% | 11.30% | 1.77% | 23.42% | 0.36% | 1.94% | 6.55% |
| Erysipelotrichales | 0.00% | 0.13% | 0.32% | 0.01% | 0.00% | 0.25% | 0.08% | 0.04% | 0.04% | 1.56% |

**Table S4.** Microbial families (presence in %) detected in *H. dromedarii* over a year.

| **Family** | **19.Mar** | **19.Apr** | **19.May** | **19.Jun** | **19.Jul** | **19.Sep** | **19.Oct** | **19.Nov** | **19.Dec** | **20.Jan** |
| --- | --- | --- | --- | --- | --- | --- | --- | --- | --- | --- |
| Actinomycetaceae | 0.00% | 0.00% | 0.00% | 0.23% | 0.00% | 0.00% | 21.09% | 0.02% | 7.00% | 0.16% |
| Corynebacteriaceae | 19.87% | 3.22% | 20.50% | 31.60% | 4.08% | 1.24% | 16.83% | 3.80% | 13.28% | 38.17% |
| Dermabacteraceae | 0.26% | 0.24% | 1.51% | 0.03% | 0.01% | 0.00% | 0.03% | 0.00% | 0.00% | 8.22% |
| Dermacoccaceae | 0.00% | 0.00% | 0.00% | 0.00% | 0.10% | 0.02% | 0.00% | 0.00% | 0.12% | 0.00% |
| Micrococcaceae | 0.58% | 0.88% | 4.80% | 7.41% | 0.03% | 0.39% | 0.54% | 0.02% | 0.29% | 6.31% |
| Rickettsiaceae | 0.00% | 0.00% | 0.00% | 0.00% | 0.00% | 2.13% | 0.00% | 0.00% | 0.00% | 0.00% |
| Neisseriaceae | 0.12% | 0.02% | 9.14% | 0.34% | 0.00% | 0.80% | 0.02% | 0.00% | 1.13% | 0.38% |
| Enterobacteriaceae | 0.19% | 33.85% | 0.14% | 1.04% | 0.05% | 0.16% | 0.14% | 0.34% | 1.20% | 0.33% |
| Moraxellaceae | 6.89% | 10.27% | 14.13% | 1.29% | 0.13% | 0.25% | 1.29% | 0.02% | 0.46% | 1.55% |
| Pseudomonadaceae | 16.59% | 2.82% | 0.42% | 0.18% | 0.00% | 0.20% | 0.07% | 0.00% | 0.00% | 0.70% |
| Francisellaceae | 10.67% | 32.86% | 12.43% | 0.50% | 17.63% | 79.42% | 18.12% | 3.94% | 9.37% | 14.66% |
| Bacillaceae | 1.12% | 0.84% | 4.74% | 8.49% | 8.80% | 1.34% | 0.57% | 0.70% | 57.00% | 2.49% |
| Planococcaceae | 0.95% | 0.30% | 2.04% | 0.72% | 0.09% | 0.27% | 0.14% | 0.00% | 0.08% | 0.97% |
| Staphylococcaceae | 39.92% | 9.08% | 13.83% | 44.18% | 53.11% | 6.24% | 4.88% | 9.66% | 2.52% | 6.17% |
| Aerococcaceae | 0.03% | 0.00% | 0.51% | 0.06% | 0.04% | 0.01% | 1.48% | 0.06% | 0.41% | 2.07% |
| Clostridiales | 0.19% | 0.00% | 0.68% | 0.09% | 11.21% | 0.00% | 11.57% | 0.08% | 1.62% | 2.60% |

**Table S5.** Microbial genera (presence in %) detected in *H. dromedarii* over a year.

| **Genus** | **19.Mar** | **19.Apr** | **19.May** | **19.Jun** | **19.Jul** | **19.Sep** | **19.Oct** | **19.Nov** | **19.Dec** | **20.Jan** |
| --- | --- | --- | --- | --- | --- | --- | --- | --- | --- | --- |
| Trueperella | 0.00% | 0.00% | 0.00% | 0.23% | 0.00% | 0.00% | 19.18% | 0.00% | 7.00% | 0.00% |
| Corynebacterium | 19.87% | 3.22% | 20.50% | 31.60% | 4.08% | 1.24% | 16.83% | 3.80% | 13.28% | 38.17% |
| Brachybacterium | 0.26% | 0.24% | 1.51% | 0.03% | 0.01% | 0.00% | 0.03% | 0.00% | 0.00% | 8.22% |
| Arthrobacter | 0.46% | 0.63% | 3.60% | 7.34% | 0.03% | 0.38% | 0.50% | 0.01% | 0.29% | 5.59% |
| Rickettsia | 0.00% | 0.00% | 0.00% | 0.00% | 0.00% | 2.13% | 0.00% | 0.00% | 0.00% | 0.00% |
| Uruburuella | 0.12% | 0.00% | 9.14% | 0.34% | 0.00% | 0.04% | 0.00% | 0.00% | 1.13% | 0.15% |
| Acinetobacter | 2.19% | 10.20% | 14.04% | 0.68% | 0.00% | 0.09% | 0.20% | 0.02% | 0.36% | 0.72% |
| Moraxella | 4.00% | 0.00% | 0.02% | 0.60% | 0.00% | 0.01% | 0.00% | 0.00% | 0.11% | 0.59% |
| Pseudomonas | 16.59% | 2.82% | 0.42% | 0.18% | 0.00% | 0.20% | 0.07% | 0.00% | 0.00% | 0.00% |
| Francisella | 10.67% | 32.86% | 12.43% | 0.50% | 17.63% | 79.42% | 18.12% | 3.94% | 9.37% | 14.66% |
| Bacillus | 1.12% | 0.84% | 4.72% | 8.49% | 8.80% | 1.34% | 0.57% | 0.70% | 57.00% | 2.49% |
| Staphylococcus | 39.79% | 8.61% | 12.53% | 44.00% | 53.10% | 6.12% | 4.83% | 9.64% | 2.52% | 6.16% |
| Anaerococcus | 0.00% | 0.00% | 0.35% | 0.00% | 9.01% | 0.00% | 6.56% | 0.00% | 0.63% | 0.71% |
| Peptoniphilus | 0.02% | 0.00% | 0.00% | 0.00% | 0.65% | 0.00% | 3.61% | 0.00% | 0.81% | 0.25% |
| Murdochiella | 0.00% | 0.00% | 0.00% | 0.00% | 0.00% | 0.00% | 7.53% | 0.00% | 0.00% | 0.01% |

**Table S6.** Correlation matrix shows pairwise Pearson’s r correlations between genera (bottom) and their associated significance (top).


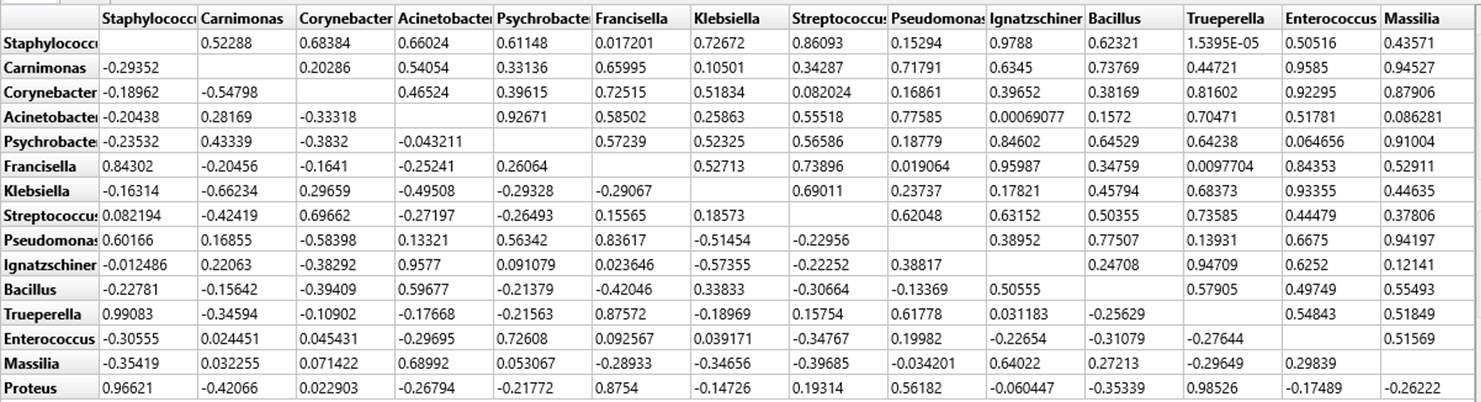

Supplement: Supplementary file 1 [file Data_Sheet_1.docx]
